# Study on dynamic alterations of plasma lipid profiles during disease progression in combined allergic rhinitis and asthma syndrome based on lipidomics

**DOI:** 10.3389/fimmu.2025.1666214

**Published:** 2025-10-22

**Authors:** Yanmin Shi, Yiting Li, Zifan Cheng, JiaJia Wang, Suyun Li, Yang Xie

**Affiliations:** ^1^ National Regional Traditional Chinese Medicine (Lung Disease) Diagnosis and Treatment Center, The First Affiliated Hospital of Henan University of Chinese Medicine, Zhengzhou, China; ^2^ Collaborative Innovation Center for Chinese Medicine and Respiratory Diseases Co-Construction by Henan Province and Education Ministry of P.R. China, Henan University of Chinese Medicine, Zhengzhou, China; ^3^ Henan International Joint Laboratory of Evidence-based Evaluation for Respiratory Diseases, Henan Province Clinical Research Center for Respiratory Diseases, The First Affiliated Hospital of Henan University of Chinese Medicine, Zhengzhou, China

**Keywords:** allergic inflammation, allergic syndrome, asthma, lipidomics, mucin

## Abstract

Combined allergic rhinitis and asthma syndrome (CARAS) involves complex interactions between inflammation and lipid metabolism. This study recruited 90 CARAS patients admitted to the First Affiliated Hospital of Henan University of Chinese Medicine from August 2023 to August 2024 (30 cases each for CARASa, CARASb and CARASc), along with 30 healthy controls (HC). We systematically profiled serum lipidomes across different CARAS stages and examined associations with inflammatory cytokines and mucins. Baseline characteristics were comparable among healthy controls (HC) and CARAS subgroups. CARAS patients in the acute phase (CARASa) exhibited elevated serum-specific IgE and fractional exhaled nitric oxide, indicating heightened allergic sensitization, while pulmonary function remained preserved. Lipidomic analysis revealed a pronounced shift from fatty acids to glycerolipids in CARASa, with upregulation of triglycerides, digalactosyldiacylglycerol, phosphatidylserines, phosphatidylethanolamines, and ceramides. CARASb (chronic persistence) showed persistent dysregulation of sphingomyelins, lysophosphatidylcholines, and membrane lipids, whereas CARASc (clinical remission) exhibited partial recovery with residual alterations in specific lipid classes. Correlation analysis indicated that fatty acid depletion strongly associated with glycerolipid accumulation. Pathway enrichment highlighted stage-dependent disturbances in fatty acid transport, GLP-1/incretin turnover, sphingolipid biosynthesis, and retinoid metabolism, reflecting metabolic-immune crosstalk. Notably, differential lipids (Digalactosyldiacylglycerol, phosphatidylethanolamines and phosphatidylserine) positively correlated with pro-inflammatory cytokines (*TNF-α*, *IL-6*) and mucins (*MUC1*, *MUC5AC*) in CARASa and CARASb groups. In the CARASc group, these differential lipids showed a negative correlation with pro-inflammatory factors and mucins. These findings define a trajectory of stage-specific lipid metabolic remodeling in CARAS, linking energy metabolism and membrane lipid changes to inflammatory activation and mucin expression, providing potential metabolic biomarkers and therapeutic targets.

## 1 Introduction

Combined Allergic Rhinitis and Asthma Syndrome (CARAS) is an allergic inflammatory disorder that simultaneously affects both the upper and lower airways, manifesting as a single syndrome across respiratory compartments (1, 2). The notion of a “united airway” underlies this concept: rhinitis and asthma share overlapping immunopathology, risk factors, and often co-occur (3, 4). In fact, intranasal corticosteroids (INCS), which target nasal inflammation, have been explored for beneficial effects on asthma outcomes in CARAS, supporting the concept of shared mucosal inflammation pathways (3).

During its pathogenesis, airway epithelial barriers respond to environmental stimuli by releasing mediators, transmitting signals, and activating innate immune cells, which in turn dysregulate a broad array of effector molecules (5). Indeed, the transition from upper airway inflammation to lower airway hyperresponsiveness may be mediated by systemic “spillover” of inflammatory signals, recruitment of immune effectors, and modulation of distant microenvironments (6, 7). With rising air pollution and global environmental changes, the incidence of CARAS has steadily increased, making it a growing public health concern (4, 8, 9).

Lipids are fundamental to cellular homeostasis, serving as structural components of membranes, signaling molecules, and energy reserves (10, 11). Lipidomics, the comprehensive analysis of lipid species within biological systems, provides a powerful platform to characterize lipid structures, abundance, and dynamics under physiological and pathological conditions (12). Abnormal lipid metabolism has been increasingly implicated in immune regulation and chronic inflammatory diseases (13). In respiratory diseases, lipid mediators such as eicosanoids, sphingolipids, and specialized pro-resolving mediators (SPMs) regulate initiation and resolution of inflammation, bronchomotor tone, and tissue remodeling (14). Recent reviews emphasize how lipid metabolism and immune function are tightly interlinked in chronic lung diseases such as asthma and COPD (15).

Importantly, because CARAS represents a systemic disorder that integrates both allergic rhinitis and asthma, lipidomic profiling may offer new insights into the shared and distinct metabolic alterations underlying this comorbidity. Indeed, in asthma research, lipidomic stratification of induced sputum has been shown to discriminate phenotypes of disease (16). Moreover, plasma lipidomic studies in asthmatic patients have already revealed altered species (e.g. phosphatidylethanolamine, sphingomyelin, triglycerides) correlated with disease severity and immunoglobulin E (IgE) levels (17). On the genomic front, lipid metabolism-related genes have been implicated in asthma pathogenesis and shown to modulate the local immune microenvironment (18).

Despite growing recognition of the immunological and inflammatory aspects of CARAS, the role of systemic lipid metabolism in its initiation, exacerbation, and remission remains poorly defined. A central unanswered question is whether lipidomic alterations can explain disease progression and heterogeneity across clinical stages of CARAS. We hypothesize that distinct lipid metabolic signatures are associated with acute exacerbation, chronic persistence, and remission of CARAS, and that these signatures may help elucidate pathogenic mechanisms and identify candidate biomarkers.

By systematically characterizing serum lipid profiles across disease stages, our study aims to: define lipid alterations linked to CARAS pathophysiology, explore their associations with inflammatory mediators and mucins, provide a metabolic framework for improved diagnosis, subtyping, and therapeutic strategies. This lipidomic perspective may ultimately contribute to a clearer understanding of CARAS as a unified airway syndrome and inform precision medicine approaches for allergic airway diseases.

## 2 Materials and methods

### 2.1 Ethical approval and informed consent

This study was approved by the Ethics Committee of the First Affiliated Hospital of Henan University of Traditional Chinese Medicine in accordance with the Helsinki Declaration (2023HL-113-01). Notify patients participating in this study of relevant information and obtain their consent and sign an informed consent form (**Supplementary Table S1**).

### 2.2 Research subjects and enrollment process

#### 2.2.1 Research subjects

30 CARASa patients (CARASa group), 30 CARASb patients (CARASb group), 30 CARASc patients (CARASc group) and 30 healthy volunteers (HC group) admitted to the First Affiliated Hospital of Henan University of Traditional Chinese Medicine from August 2023 to August 2024 were selected as the study subjects. Collect basic and main clinical information of three groups of research subjects, including basic information (age, gender, BMI, smoking history and drinking history) and clinical data (blood routine). The basic information and main clinical information of all research subjects are shown in **Table 1**.

**Table 1:** Clinical data of patients with combined allergic rhinitis and asthma syndrome at different stages.

Variables	HC group	CARASa group	CARASb group	CARASc group	*P-*value
Gender (Male)	10 (33.3%)	10 (33.3%)	10 (33.3%)	11 (36.7%)	NA
Age-years	42.83 ± 16.13	44.80 ± 11.66	46.73 ± 14.28	40.13 ± 10.44	0.37
BMI	23.10 ± 2.92	25.10 ± 5.80	24.49 ± 3.78	24.25 ± 3.17	0.56
Smoking history	2 (6.7%)	4 (13.3%)	2 (6.7%)	3 (10.0%)	NA
Drinking history	2 (6.7%)	4 (13.3%)	2 (6.7%)	4 (13.3%)	NA
Blood routine					
WBC	5.72 ± 1.26	6.57 ± 2.02	6.28 ± 1.70	6.12 ± 1.67	0.28
RBC	4.70 ± 0.46	4.65 ± 0.48	4.74 ± 0.50	4.54 ± 0.61	0.62
PLT	240.73 ± 56.74	247.13 ± 68.48	229.73 ± 56.64	229.52 ± 70.89	0.39
NEU	3.46 ± 1.07	5.41 ± 7.81	3.81 ± 1.16	3.61 ± 1.15	0.48
EOS	0.12 ± 0.10	0.44 ± 0.47	0.29 ± 0.37	0.29 ± 0.25	0.19
IgE	150.11 ± 37.37^c^	4454.88 ± 565.20^a^	78.38 ± 32.86^c^	228.65 ± 26.61^b^	< 0.05
Respiratory Function					
FVC	3.84 ± 0.99	3.69 ± 0.73	3.93 ± 1.23	3.78 ± 0.96	0.27
FEV1	3.23 ± 0.90	2.58 ± 0.73	2.83 ± 1.23	2.82 ± 0.96	0.35
FEV1/FVC	84.77 ± 9.53	69.61 ± 11.92	73.35 ± 8.10	74.91 ± 8.91	0.15
FeNO	14.33 ± 4.55^d^	87.92 ± 37.10^a^	22.90 ± 2.47^c^	48.68 ± 6.34^b^	< 0.05

WBC, White Blood Cell; RBC, Red Blood Cell; PLT, Platelet; NEU, Neutrophil; EOS, Eosinophil; lgE, Immunoglobulin E; FVC, Forced Vital Capacity; FEV1, Forced Expiratory Volume in 1 second; FEV1/FVC, Forced Expiratory Volume in 1 second/Forced Vital Capacity; FeNO, Fractional Exhaled Nitric Oxide. Data in the same row are marked with different capitals a, b and c, indicating significant difference (*P-*value < 0.05).

#### 2.2.2 Enrollment process

Inclusion criteria: (1) Diagnosis of allergic rhinitis: Met symptom criteria per Allergic Rhinitis and its Impact on Asthma (ARIA) guidelines (nasal itching, sneezing, rhinorrhea, congestion ≥4 days/week for ≥4 weeks); Positive allergen test (skin prick test or serum IgE confirming sensitization to aeroallergens). (2) Diagnosis of bronchial asthma: Met Global Initiative for Asthma (GINA) diagnostic criteria (reversible airflow limitation: post-bronchodilator FEV_1_ improvement ≥ 12% and absolute increase ≥ 200 mL); Typical asthma symptoms. (3) Temporal association: Clear time-linked exacerbation (e.g., rhinitis episodes triggering/worsening asthma); Consistent allergen sensitization profiles for both conditions. (4) Age: 12–65 years; disease duration of asthma or allergic rhinitis ≥1 year.

Exclusion criteria: (1) Other respiratory/Nasal disorders: Chronic rhinosinusitis with nasal polyps, non-allergic rhinitis (e.g., vasomotor rhinitis); Chronic obstructive pulmonary disease, bronchiectasis, pulmonary fibrosis, active tuberculosis. (2) Systemic comorbidities: Severe cardiac/hepatic/renal failure; Immunodeficiency disorders, active malignancies. (3) Recent interventions: Systemic glucocorticoids or immunosuppressants within 4 weeks; Allergen immunotherapy within 6 months. (4) Special populations: Pregnancy or lactation; Inability to cooperate (e.g., severe cognitive impairment, psychiatric disorders).

### 2.3 Sample collection

Using sterile disposable vacuum blood collection needles and EDTA-anticoagulated tubes, perform venipuncture for blood collection and immediately conduct complete blood count analysis. Subsequently, collect a portion of the plasma sample for subsequent lipid identification. Collect a peripheral blood sample and isolate peripheral blood mononuclear cells (PBMCs) through Ficoll density gradient centrifugation, followed by cryopreservation in liquid nitrogen for subsequent inflammatory factor (TNF-α and IL-6) mRNA analysis. Collect induced sputum samples for subsequent mucin (MUC5AC and MUC1) mRNA analysis.

### 2.4 Serum lipidomics analysis

#### 2.4.1 Lipid extraction

Lipids were extracted using a modified Bligh-Dyer method. 100 μL plasma was mixed with 1 mL chloroform-methanol (2:1, v/v) containing internal standards, vortexed for 5 min and centrifuged at 12, 000 ×*g* for 10 min at 4 °C. The lower organic phase was collected, dried under a nitrogen stream, reconstituted in 100 μL acetonitrile-methanol (6:4, v/v) with 0.1% formic acid, filtered through a 0.22 μm membrane and prepared for instrumental analysis.

#### 2.4.2 Lipid separation and identification (UPLC-MS)

Chromatographic conditions: Waters ACQUITY UPLC BEH C18 column (1.7 μm, 2.1×100 mm); column temperature 45 °C; flow rate 0.4 mL/min; injection volume 2 μL. Mobile phase A: methanol-water (95:5, v/v) with 0.1% formic acid; mobile phase B: acetonitrile-isopropanol (60:40, v/v) with 0.1% formic acid. Gradient program: 0–1 min, 50% B; 1–10 min, 50% to 95% B; 10–12 min, 95% B; 12–13 min, 95% to 50% B; 13–15 min, 50% B. Mass spectrometry conditions: ESI ion source with positive/negative ion switching; capillary voltage 3.0 kV; cone voltage 40 V; desolvation gas temperature 500 °C; flow rate 800 L/h; scan range m/z 100-1500.

#### 2.4.3 Data processing and analysis

Raw data were processed using Waters MassLynx 4.2. Lipid annotation was performed via LipidSearch 4.1, with structural confirmation against the LIPID MAPS database and authentic standards. Quantitation used internal standardization. Differential lipids were screened by multivariate statistics (PLS-DA) with significance thresholds of *P* < 0.05 and variable importance in projection (VIP) >1.0.

### 2.5 qRT-PCR

Total RNA was isolated following the manufacturer’s instructions and quantified with a NanoDrop ND-2000 spectrophotometer (Thermo Fisher Scientific, Waltham, MA, USA). The RNA integrity of each sample was evaluated via denaturing agarose gel electrophoresis (Liuyi Biotechnology, Beijing, China). In brief, 1 μg of total RNA was subjected to treatment with an RNase-free DNase I set, after which reverse transcription was conducted using a ReverAid First Strand cDNA Synthesis kit (Thermo Scientific, USA). Subsequently, qRT-PCR analysis was carried out using a LightCycler 96 instrument (Roch, USA) along with SYBR Green master mix (BioRed, USA). The GAPDH gene served as the reference gene for normalization, and the results were calculated using the 2-ΔΔCt method. Detailed information on genes and primers is provided in **Supplementary Table S1**. For qRT-PCR analysis, three biological replicates were included.

### 2.6 Statistical analysis

All data were processed using SPSS 24.0 statistical software. One way analysis of variance (ANOVA) was used for data that followed a normal distribution, while non parametric tests (Kruskal Wallis) were used for data that did not follow a normal distribution. *P* < 0.05 was considered statistically significant.

## 3 Results

### 3.1 Clinical characteristics indicate stage-dependent inflammatory activation

The baseline characteristics (age, gender, BMI, smoking and drinking history) were comparable among HC and CARAS subgroups (**Table 1**). However, CARASa patients displayed significantly elevated serum-IgE and fractional exhaled nitric oxide (FeNO) levels compared with HC and other CARAS subgroups (*P* < 0.05). These findings suggest stronger allergic sensitization and eosinophilic inflammation during acute exacerbation, whereas pulmonary function parameters (FVC, FEV1/FVC, FEV1%) remained preserved across groups (*P* > 0.05). This indicates that inflammatory and metabolic dysregulation may precede overt functional decline.

### 3.2 Global lipidomic profiling reveals a consistent shift from fatty acids to glycerolipids

**Figure 1:**
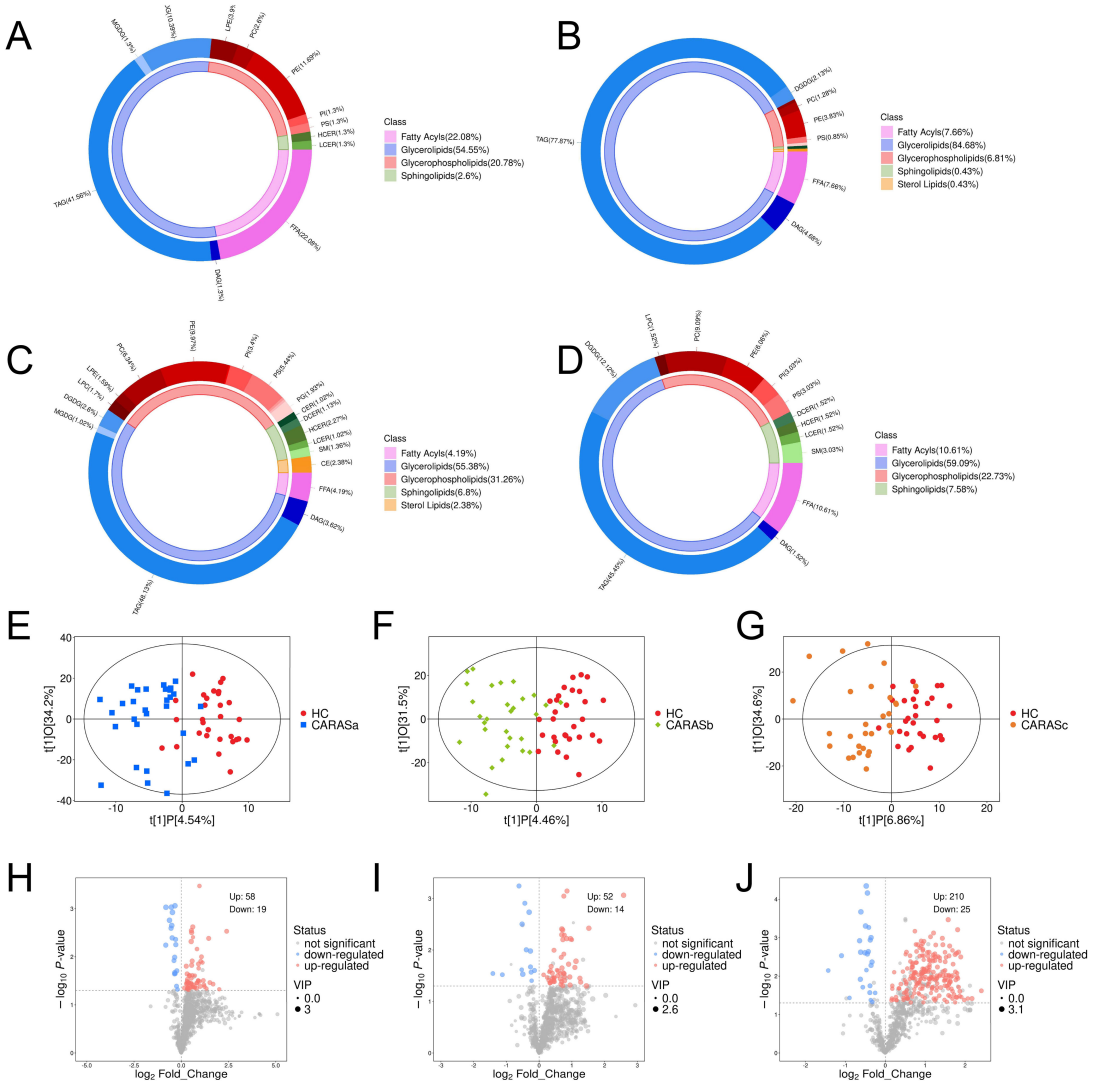
Lipid composition and differential lipid analysis. **(A)** Plasma lipid composition in HC group. **(B)** Plasma lipid composition in CARASa group. **(C)** Plasma lipid composition in CARASb group. **(D)** Plasma lipid composition in CARASc group. **(E)** Scatter plot of OPLS-DA scores between CARASa and HC group. **(F)** Scatter plot of OPLS-DA scores between CARASb and HC group. **(G)** Scatter plot of OPLS-DA scores between CARASc and HC group. **(H)** Differential lipid volcano plot between CARASa and HC group. **(I)** Differential lipid volcano plot between CARASb group and HC group. **(J)** Differential lipid volcano plot between CARASc and HC group.

Lipid compositional analysis revealed a marked redistribution of lipid categories across CARAS subgroups. Compared with HC (FA 22.08%, GL 54.55%), CARASa showed a striking depletion of fatty acids (FA, 7.66%) and expansion of glycerolipids (GL, 84.68%), while CARASb and CARASc demonstrated similar but stage-modulated patterns (**Figures 1A-D**). OPLS-DA score plots confirmed distinct separation between HC and CARAS groups, with > 95% of samples within Hotelling’s T-squared ellipses (**Figures 1E-G**). Volcano plots identified progressively larger sets of differentially abundant lipids from CARASa (total: 77, upregulated: 58, downregulated: 19) to CARASb (total: 66, upregulated: 52, downregulated: 19) and CARASc (total: 235, upregulated: 210, downregulated: 25) versus HC (**Figures 1H-J**). As the lipid annotations were primarily database-driven with limited use of authentic standards, the identifications should be regarded as putative and the quantification as semi-quantitative, underscoring the inherent limitations of untargeted lipidomics.

### 3.3 Differential metabolite signatures define stage-specific lipid remodeling

**Figure 2:**
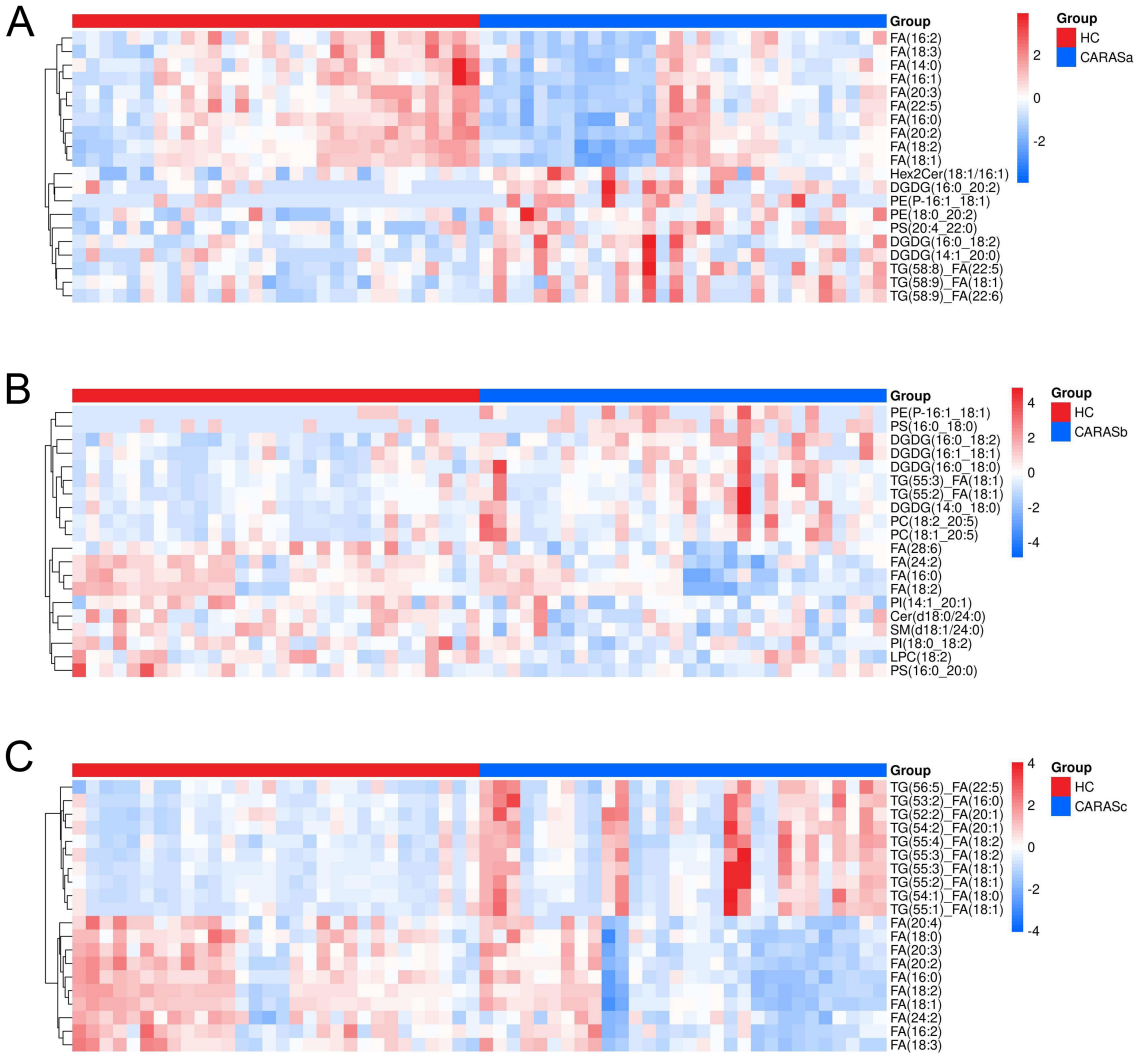
Hierarchical clustering of differential lipids. **(A)** Hierarchical clustering analysis of differential lipids between CARASa and HC groups (Note: The horizontal axis represents experimental groups, while the vertical axis displays differential metabolites for the group comparison. Color intensity in each tile indicates the relative abundance of the corresponding metabolite: red denotes higher abundance in its respective group, and blue indicates lower abundance). **(B)** Hierarchical clustering analysis of differential lipids between CARASb and HC groups. **(C)** Hierarchical clustering analysis of differential lipids between CARASc and HC groups.

**Figure 3:**
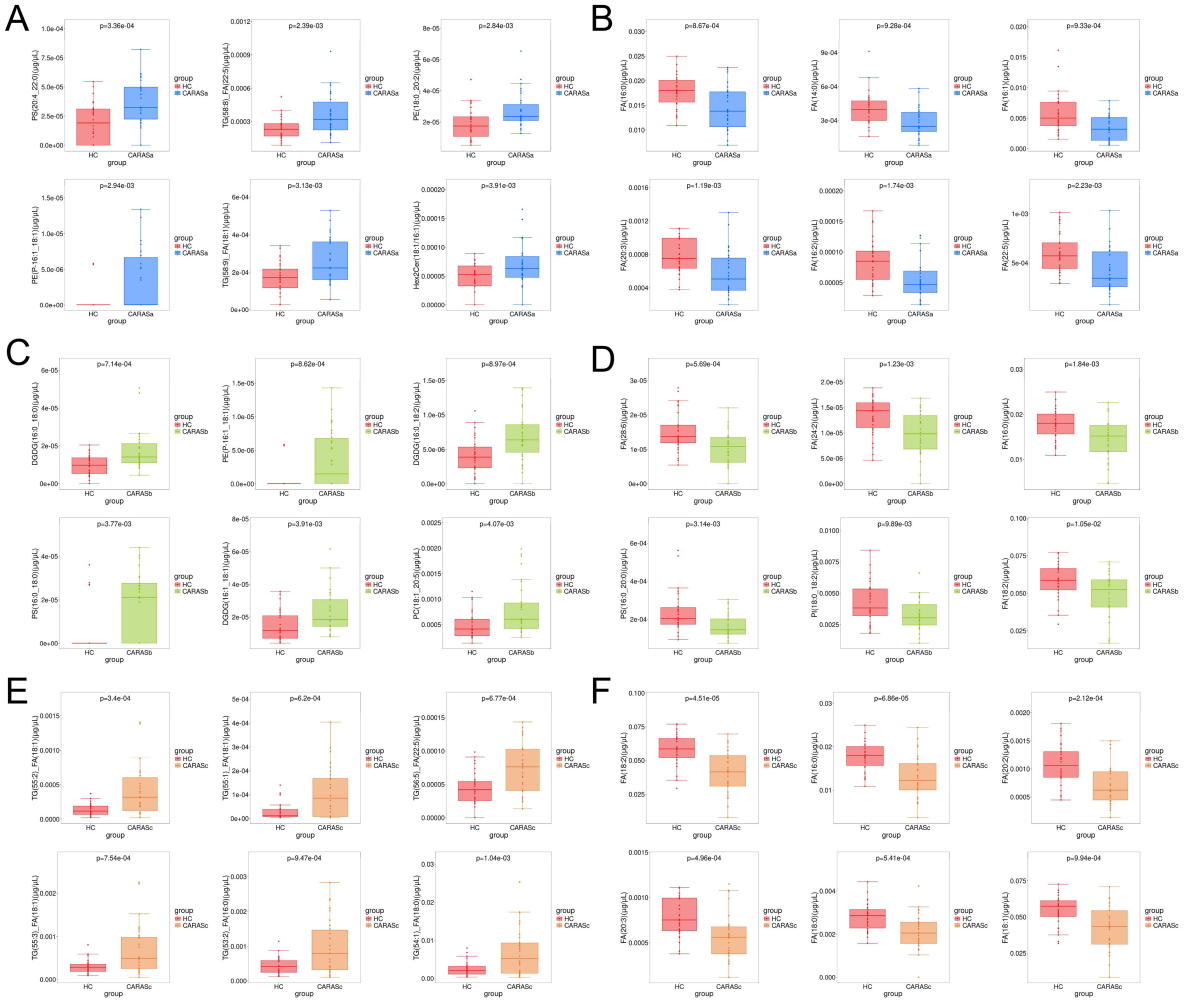
Box-plot analysis of differential lipids. **(A)** Lipids significantly upregulated in CARASa compared with HC group. **(B)** Lipids significantly downregulated in CARASa compared with HC group. **(C)** Lipids significantly upregulated in CARASb compared with HC group. **(D)** Lipids significantly downregulated in CARASb compared with HC group. **(E)** Lipids significantly upregulated in CARASc compared with HC group. **(F)** Lipids significantly downregulated in CARASc compared with HC group.

Lipidomic profiling revealed distinct metabolic signatures across different clinical stages of CARAS (**Figures 2**, **3**). Differential lipid analysis and hierarchical clustering further illustrated stage-specific patterns. Specifically, the results demonstrated a clear separation between CARASa (acute exacerbation) and healthy controls, indicating pronounced lipid disturbances during the acute phase. CARASa was characterized by a significant upregulation of lipid species previously implicated in inflammatory responses, including phosphatidylserine (PSs), phosphatidylethanolamines (PEs), ceramides (Hex2Cers), digalactosyldiacylglycerol (DGDGs) and triglycerides (TGs), consistent with acute immune activation and membrane remodeling. CARASb (chronic persistence) still exhibited a marked deviation from controls, though less pronounced than CARASa, persistent dysregulation was observed mainly in sphingomyelins (SMs), lysophosphatidylcholines (LPCs), phosphatidylserine (PSs), phosphatidylethanolamines (PEs) and digalactosyldiacylglycerol (DGDGs), reflecting chronic metabolic imbalance and impaired lipid signaling. In contrast, PEs, PSs, and DGDGs were significantly downregulated in the CARASc group compared with the HC group.

### 3.4 Correlation analysis links FA depletion to glycerolipid accumulation

**Figure 4:**
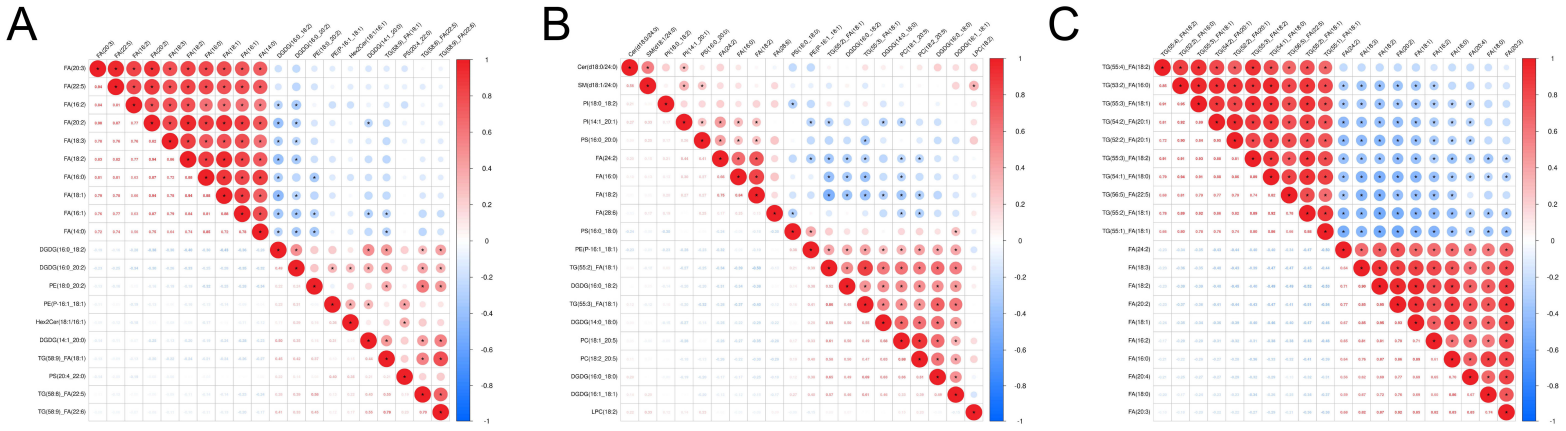
Correlation analysis and lipid composition changes. **(A)** Correlation analysis heatmap between CARASa and HC group (Note: The horizontal and vertical axes represent differential metabolites for the group comparison. Each color of square indicates the correlation coefficient magnitude between corresponding metabolites: red signifies positive correlation, blue denotes negative correlation, and color intensity scales with correlation strength. Asterisks (*) mark statistically significant correlations *P* < 0.05). **(B)** Correlation analysis heatmap between CARASb and HC group. **(C)** Correlation analysis heatmap between CARASc and HC group.

**Figure 5:**
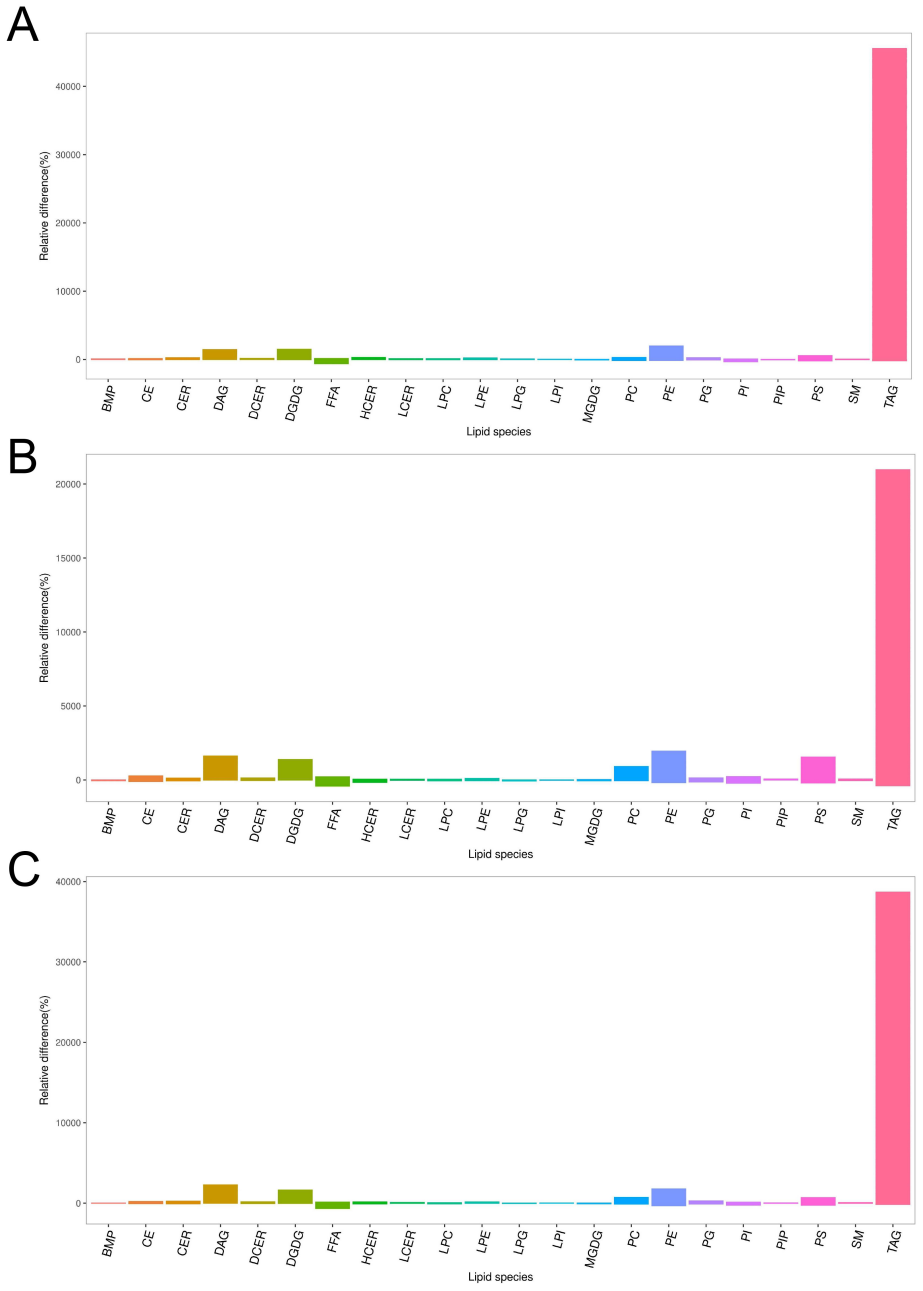
Column chart of lipid composition between CARAS groups and HC group. **(A)** Column chart of lipid composition between CARASa group and HC group (Note: In the lipidomics bar chart, each bar represents a class of metabolites. The ordinate of the chart represents the percentage of relative change in the content of each substance in the comparison of this group. If the percentage of relative change in content is zero, it indicates that the content of the substance is the same in both groups; a positive percentage of relative change in content indicates that the content of the substance is higher in the CARASa group; a negative percentage of relative change in content indicates that the content of the substance is higher in the HC group. The abscissa of the lipidomics bar chart indicates the classification information of lipids). **(B)** Column chart of lipid composition between CARASb group and HC group. **(C)** Column chart of lipid composition between CARASc group and HC group.

Correlation heatmaps revealed strong negative correlations between FA and several glycerolipids, including DGDG, PE, and TG (**Figures 4A-C**). Bar plots further confirmed the consistent elevation of these glycerolipids (DGDG, PE, and TG) across CARAS stages relative to HC (**Figures 5A-C**). Collectively, the data show that depletion of FA pools is accompanied by a metabolic flux shift toward glycerolipid accumulation.

### 3.5 Pathway enrichment highlights lipid transport and metabolic-immune crosstalk

**Figure 6:**
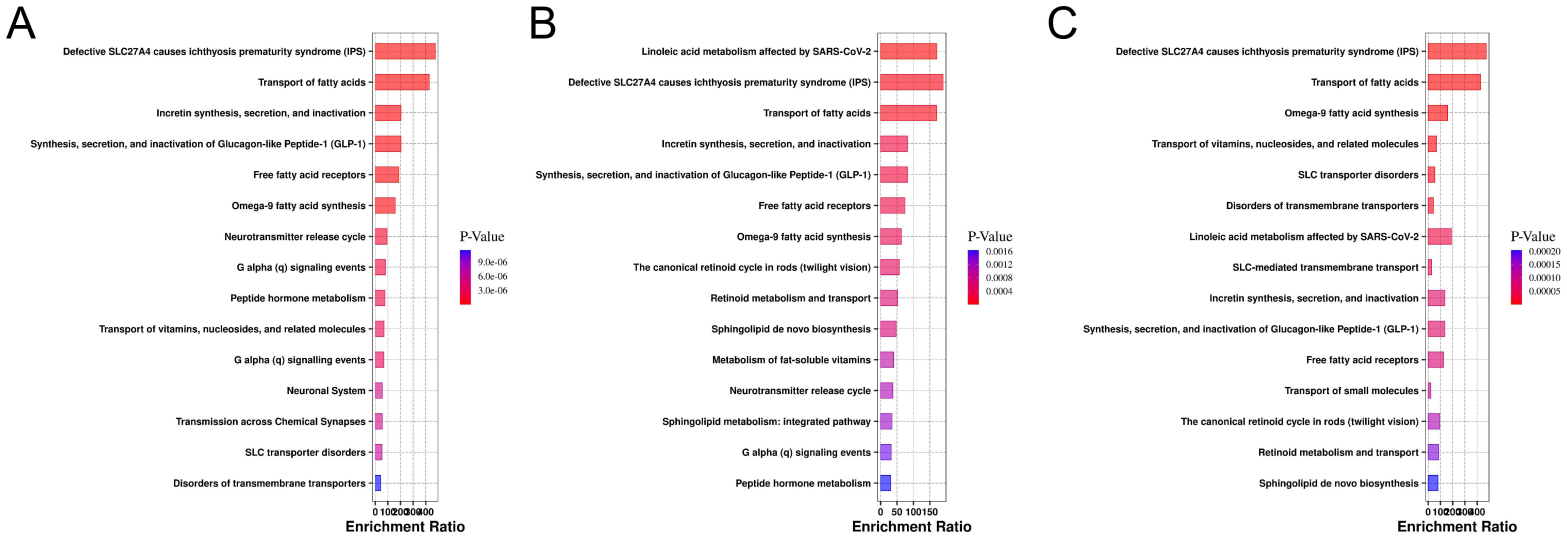
Enrichment analysis of metabolic pathways for differential lipids in the database. **(A)** Pathway enrichment plot of the CARASa group compared with the HC group (The x-axis represents the enrichment ratio for each pathway, while the y-axis lists metabolic pathway names. Color intensity indicates the magnitude of the P-value: smaller P-values correspond to redder hues, denoting greater enrichment significance). **(B)** Pathway enrichment plot of the CARASb group compared with the HC group. **(C)** Pathway enrichment plot of the CARASc group compared with the HC group.

In this study, we systematically analyzed lipid metabolic pathways across different clinical stages of CARAS. Several core pathways, including fatty acid transport and signaling related to free fatty acid receptors and GLP-1/incretin function, remained consistently enriched across all stages. In the acute phase, differential lipids were mainly associated with fatty acid transport, GLP-1 and incretin turnover, omega-9 fatty acid synthesis, and neurotransmitter release, suggesting a tight link between acute inflammation, energy metabolism, and neuroimmune regulation (**Figure 6A**). During the chronic persistent phase, the metabolic network became more complex, with additional enrichment in linoleic acid metabolism, retinoid cycling and transport, sphingolipid biosynthesis, and fat-soluble vitamin metabolism, reflecting a shift toward structural and vitamin-related disturbances under sustained inflammation (**Figure 6B**). In the clinical remission phase, the lipid metabolic profile showed partial recovery, however, persistent alterations in sphingolipid and retinoid pathways indicated incomplete normalization (**Figure 6C**). Collectively, CARAS progression is marked by a transition from acute energy and signaling disruption to broader metabolic imbalance, followed by partial but unresolved recovery.

### 3.6 Correlation analysis reveals associations between differential lipids, inflammatory cytokines, and mucins

**Figure 7:**
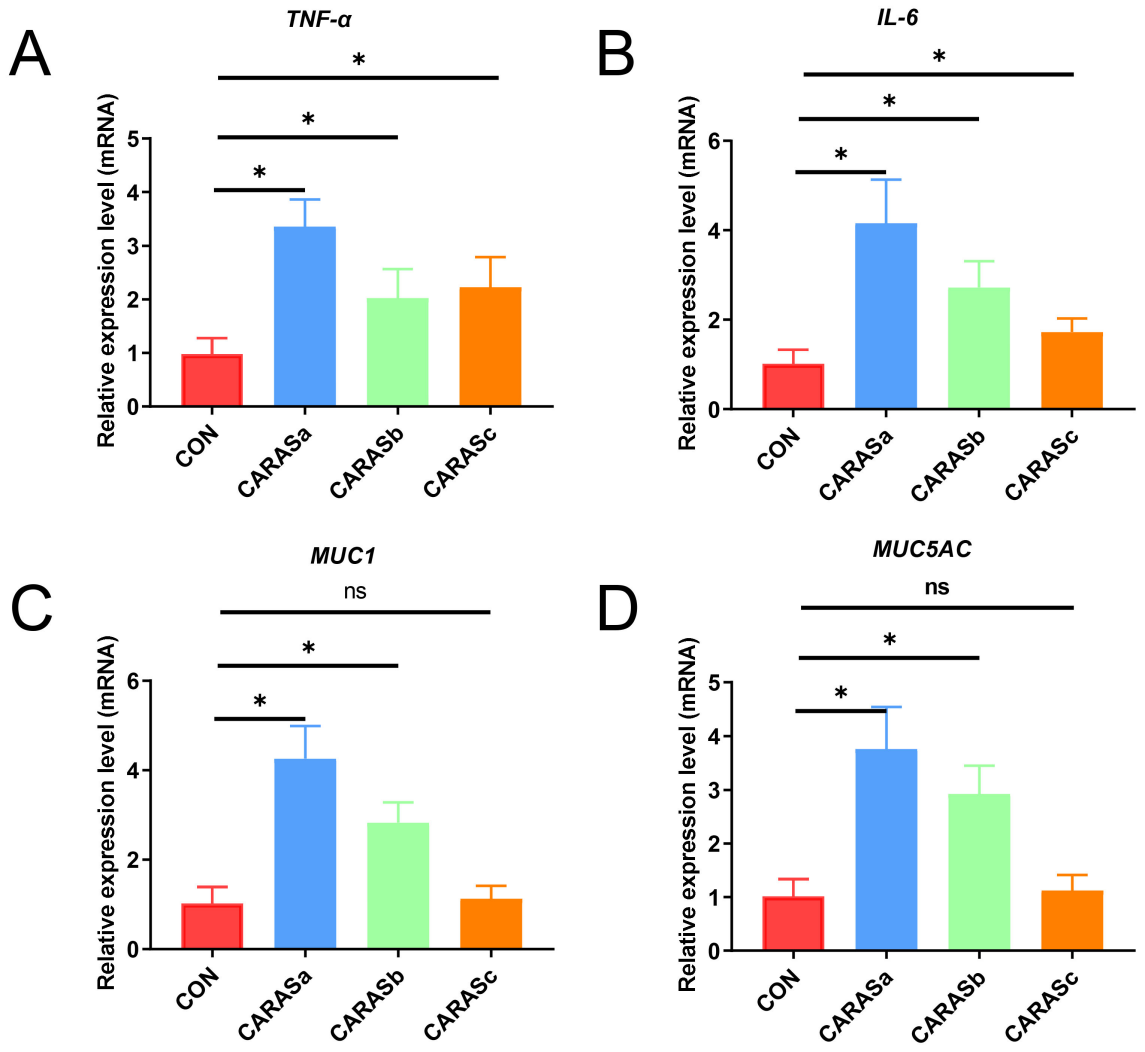
Analysis of relative expression levels of inflammatory cytokines and mucins. **(A, B)** Relative expression levels of inflammatory cytokines (*TNF-α* and *IL-6*) in blood. **(C, D)** Relative expression levels of mucins (*MUC5AC* and *MUC1*) in sputum. n = 10. * *P* < 0.05 was considered statistically significant. ns *P* > 0.05 was considered no statistically significant.

**Figure 8:**
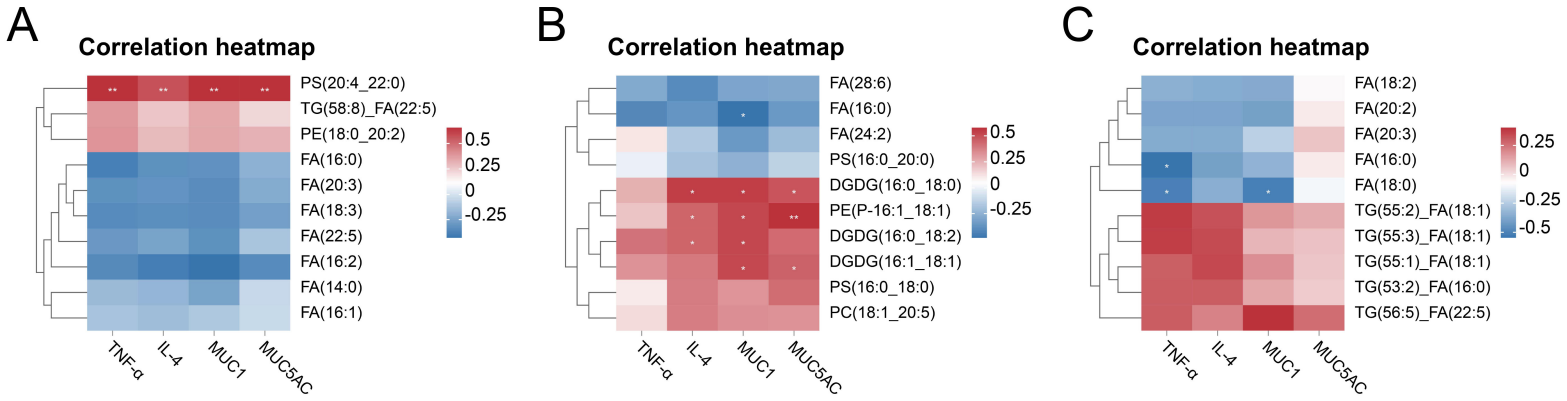
Correlation analysis between differential lipids and inflammatory cytokines, mucins. **(A)** Correlation heatmap of CARASa group and HC group. **(B)** Correlation heatmap of CARASb group and HC group. **(C)** Correlation heatmap of CARASc group and HC group. *P < 0.05 and **P < 0.01 were considered statistically significant.

qPCR validation demonstrated significant upregulation of pro-inflammatory cytokines (*TNF-α*, *IL-6*) in all CARAS groups compared with HC (*P* < 0.05). Mucin expression (*MUC1* and *MUC5AC*) was significantly increased in CARASa and CARASb (*P* < 0.05), but not CARASc (*P* > 0.05) (**Figure 7**). Correlation analysis of inflammatory factors and mucins with differential lipid profiles showed that in the CARASa and CARASb groups, inflammatory factors (*TNF-α* and *IL-6*) and mucins (*MUC1* and *MUC5AC*) were positively correlated with differential lipid profiles (PSs and PEs), but negatively correlated with FAs. In the CARASc group, inflammatory factors (*TNF-α* and *IL-6*) and mucins (*MUC1* and *MUC5AC*) were positively correlated only with differential lipid profiles (TGs), but negatively correlated with FAs (**Figure 8**).

## 4 Discussion

In this study, we systematically characterized lipid metabolic alterations in patients with combined allergic rhinitis and asthma syndrome (CARAS) across different clinical stages, and integrated these findings with inflammatory cytokines and mucin expression. Several novel insights were obtained that may contribute to understanding the interplay between lipid metabolism, immune activation, and epithelial barrier dysfunction in CARAS.

The lipidomic analysis revealed that triglycerides (TG), diacylglycerols (DG), and free fatty acids (FA) were significantly elevated in CARAS patients compared with healthy controls. These lipids showed a strong positive correlation with IgE levels and disease severity, suggesting that enhanced lipid mobilization may accompany or even promote allergic inflammation. Similar associations have been reported in allergic rhinitis and asthma cohorts, where TG, DG, and FA concentrations were linked to exacerbation severity and immunoglobulin responses (17, 19). The accumulation of these neutral lipids may contribute to energy demands during chronic inflammation and facilitate the production of pro-inflammatory lipid mediators.

In addition, we observed marked alterations in glycerophospholipids (GPs) and sphingolipids (SPs), particularly phosphatidylethanolamine (PE) and phosphatidylserine (PS). Enrichment analysis indicated significant involvement of the glycerophospholipid and sphingolipid pathways. These pathways have been previously implicated in airway remodeling and immune cell activation in both murine models of allergic sensitization and clinical asthma (16, 20, 21). Given the role of phospholipids in membrane dynamics and signal transduction, disturbances in these pathways may affect epithelial integrity and leukocyte recruitment.

The pathway enrichment analysis revealed stage-dependent perturbations in lipid metabolism that reflect the dynamic immune-metabolic landscape of CARAS. In the acute phase (CARASa), enrichment of fatty acid transport, incretin turnover, and neurotransmitter-related pathways suggests that acute inflammation rapidly reprograms lipid metabolism to support energy demands and neuroimmune communication. This finding is consistent with previous studies showing that acute allergic inflammation promotes fatty acid mobilization and signaling through G-protein-coupled free fatty acid receptors, which in turn amplify cytokine responses and epithelial barrier dysfunction (22–24) (25, 26). In contrast, the chronic phase (CARASb) was characterized by broader metabolic remodeling, with additional enrichment of linoleic acid metabolism, retinoid cycling, and sphingolipid biosynthesis. These pathways are known to modulate epithelial integrity, T helper cell polarization, and mucus hypersecretion during sustained airway inflammation (27–32). Although partial recovery of lipid metabolic balance was observed during remission (CARASc), persistent alterations in sphingolipid and retinoid metabolism suggest that metabolic-immune cross-talk remains incompletely resolved, potentially underlying susceptibility to recurrent exacerbations (29, 33, 34).

Correlation analyses further emphasized the close relationship between lipid alterations, inflammatory mediators, and mucus production. In both acute and chronic phases, phosphatidylserines (PSs) and phosphatidylethanolamines (PEs) positively correlated with pro-inflammatory cytokines (TNF-α, IL-6) and mucins (MUC1, MUC5AC), while free fatty acids (FAs) exhibited an inverse correlation, indicating a pro-inflammatory role of membrane phospholipid remodeling. This aligns with evidence that phospholipid derivatives can act as signaling mediators to activate NF-κB pathways and stimulate mucin expression in airway epithelial cells (26, 34). Interestingly, in the remission phase, triglycerides (TGs) rather than phospholipids were positively associated with inflammatory mediators, suggesting a metabolic shift in the residual inflammatory state (33, 35). These findings indicate that distinct lipid species differentially interact with immune effectors depending on disease stage, highlighting lipid metabolism as both a driver and a potential biomarker of airway inflammation in CARAS.

## 5 Conclusions

This study systematically characterized lipid metabolic alterations across different clinical stages of combined allergic rhinitis and asthma syndrome (CARAS). Neutral lipids (TGs and DGs) and membrane lipids (GPs, SPs, particularly PS and PE) exhibited significant changes that were associated with disease severity, inflammatory cytokines, and mucin expression. Pathway analysis indicated stage-dependent patterns: acute inflammation was linked with fatty acid mobilization and neuroimmune signaling, chronic persistence showed alterations in linoleic acid, retinoid, and sphingolipid pathways, and remission demonstrated partial metabolic recovery. Associations between specific lipid species and inflammatory mediators were observed, suggesting a potential role of lipid metabolism in immune and epithelial responses. These results provide a detailed reference for understanding lipid-immune-epithelial interactions in CARAS and may inform future studies on stage-specific biomarkers and therapeutic strategies.

## Data Availability

The original contributions presented in the study are included in the article/**Supplementary Material**, further inquiries can be directed to the corresponding author/s.

## Glossary

CARAS: Combined Allergic Rhinitis and Asthma Syndrome
ARIA: Allergic Rhinitis and its Impact on Asthma
GINA: Global Initiative for Asthma
KEGG: Kyoto encyclopedia of genes and genomes
PBMCs: Peripheral blood mononuclear cells
ESI: Electric spray ionization source
TNF-α: Tumor necrosis factor α
IL-6: Interleukin
MUC: Mucin
IgE: Immunoglobulin E
FVC: Forced vital capacity
FEV1: Forced expiratory volume in 1 second
FA: Fatty acids
GL: Glycerolipids
GP: Glycerophospholipids
SP: Sphingolipids
ST: Sterol lipids
SL: Saccharolipids
PK: Polyketides
OPLS-DA: Orthogonal projections to latent structures discriminant analysis
DGDG: Digalactosyldiacylglycerol
TG: Triacylglycerol
DAG: Diacylglycerol
PE: Phosphatidylethanolamine
TG: Triacylglycerol
IPS: Ichthyosis prematurity syndrome
