# Supplementary material for: Study on dynamic alterations of plasma lipid profiles during disease progression in combined allergic rhinitis and asthma syndrome based on lipidomics
Source: Front Immunol. 2025 Oct 22;16:1666214. doi: 10.3389/fimmu.2025.1666214 (PMC12586177; doi:10.3389/fimmu.2025.1666214)
Supplement: Supplementary file 1 [file Table1.docx]

**Informed Consent Form for Clinical Research**

Dear Participant,

You are invited to take part in a scientific research study titled "Preliminary Application and Evaluation of Exhaled Breath Condensate Biomarkers in CARAS." The principal investigator is Shi Yanmin, and the department responsible is the Respiratory Department. The contact number is 18838906711. This project is part of the Henan Provincial Traditional Chinese Medicine Scientific Research Special Project, with the project number 2023JDZX058. This informed consent form will provide you with the following information: why this study is being conducted, what the study involves, the potential benefits you may receive, and the risks you may face. If you have any questions, please feel free to ask. You can decide whether to participate in this study after fully understanding its content.

1. Introduction: Why are we inviting you to participate in this study?

You are invited to participate in this study because you have a family history of allergic rhinitis and/or asthma (or you have been diagnosed with Combined Allergic Rhinitis and Asthma Syndrome, CARAS). Approximately 90 participants are expected to take part in this study.

1. Am I required to participate in this study?

Your participation in this study is entirely voluntary. You are free to refuse participation, and this will not affect your routine treatment. If you decide to participate, you may also withdraw from the study at any time.

1. Why is this study being conducted?

The purpose of this study is to explore the differences in biomarker expression between healthy individuals and different disease subgroups across various sample types, aiming to identify simpler, more direct, and objective potential biomarkers. Additionally, the study seeks to identify biomarkers associated with Traditional Chinese Medicine (TCM) syndromes and different stages of CARAS, providing a reference for personalized diagnosis and treatment.

1. How will the study be conducted?

This study adopts a cross-sectional survey design, enrolling 90 patients with CARAS (30 in the acute phase, 30 in the chronic phase, and 30 in the clinical control phase). Additionally, 30 healthy non-smokers will be included as a control group. Data collected will include general patient information, Asthma Control Test (ACT) scores, and Total Nasal Symptom Score (TNSS). For patients whose TCM syndromes correspond to lung qi deficiency, phlegm-heat obstructing the lung, or wind-heat invading the lung, TCM syndrome-related data will also be collected. On the same day, exhaled breath condensate (EBC), peripheral venous blood, and sputum samples will be collected for biomarker analysis. Based on the research data, variance analysis and the SNK method will be used to analyze differences in biomarker expression (e.g., interleukin-4, IL-4) across different sample types between healthy individuals and different disease subgroups. The study aims to identify potential specific biomarkers for CARAS and explore the correlation between EBC biomarkers and TCM syndrome indicators through correlation analysis, preliminarily assessing the application value of EBC biomarkers in the integration of disease and syndrome in CARAS, thereby providing a basis for personalized diagnosis and treatment.

1. How long will my participation in the study last?

Your participation in the study will last approximately half a day.

1. Can I stop participating in the study?

You may withdraw from the study at any time by notifying the researcher.

The researcher may also terminate your participation at any time for any reason and will inform you accordingly.

1. What risks might I face during the study?

This study is safe. However, if you experience any discomfort, changes in your condition, or any unforeseen circumstances during the study, whether related to the study or not, please notify your doctor promptly. He/she will assess the situation and provide medical treatment. Participation in this study may require you to undergo physical and chemical tests at the hospital, which may cause inconvenience or discomfort.

Another risk is the potential breach of confidentiality. We will take all necessary measures to protect your research records.

1. What potential benefits might I gain?

This study may not directly benefit you, but we hope that the information obtained will help us develop better treatment methods for other patients with similar conditions.

You will receive quality medical care during the study.

1. Do I need to pay to participate in this study?

You will not be required to bear any costs for participating in this study.

1. Will I receive compensation for participating in this study?

To compensate for any inconvenience caused by your participation, this study will cover the costs of blood tests, lung function tests, sputum sample tests, and EBC collection and analysis.

1. Can I receive compensation if I am harmed due to participation in this study?

No compensation fund has been established. However, if you believe you have suffered harm due to participation in the study, please notify the researcher immediately. Signing this informed consent form does not mean you waive your right to claim compensation.

1. Who should I contact if I have questions?

If you have any questions related to this study, please contact the researcher directly.

If you have any concerns about your rights as a participant, or if you have any complaints or suggestions, please contact the Ethics Committee Office.

1. What medical information will we collect?

We need your permission to collect certain medical information; otherwise, you cannot participate in the study. As described below, this information may come from our questions, forms you fill out, or your medical records. We will only collect information necessary for the study.

For example, questionnaires used in the study.

1. Who will have access to your medical information?

Only personnel involved in the clinical research at this hospital, members of the Ethics Committee, and individuals authorized to monitor compliance with hospital regulations will have access to your medical information.

We will record your medical information at this hospital, including test results, examination results, and research questionnaire outcomes. Therefore, this information may be reviewed by authorized personnel for your treatment. It may also be accessed by individuals to whom you have given written consent or as permitted by law.

We will not use this information for other studies without your permission or the approval of the Ethics Committee.

Once your identity is removed from the information, it may be used for other purposes without further consultation. The study results will be published (in public presentations or written form), but any information that could identify you will not be included.

1. What are the risks of sharing medical information?

One risk of participating in this study is that more people may have access to your medical information. The research team will make every effort to protect your information, but there is a possibility that unauthorized individuals may access it. This could cause embarrassment or affect your ability to obtain health insurance. You may discuss with the principal investigator whether you are at risk in this regard.

1. How long will my medical information be stored?

We will store this information for 5 years (or indefinitely) for future reference.

1. Can I revoke my prior consent to share medical information?

If you change your mind and do not wish for us to collect and share your information, please contact the principal investigator. At that point, we will decide whether to terminate your participation in the study, but we may continue to use the information already collected about you.

Consent to Participate in Research

Before signing, I confirm the following facts:

- I have read (or have had read to me) this entire consent document. All my questions have been satisfactorily answered.

The researcher has explained to me the purpose, procedures, and potential benefits and risks of this study.

- I consent to the research team using and sharing my medical information and other information collected from this study.
- I voluntarily agree to participate in this study. I agree to follow the study procedures as required.
- I have been informed that I may withdraw from the study at any time.

Please Note: You will receive a copy of this signed and dated consent form. Please keep this form in a safe and easily accessible place, as it will help you remember what we have discussed today.

Subject Signature: ________________________

Date: _______ Year ___ Month ___ Day

Subject's Contact Number: ________________________

Mobile Number: ________________________

Legal Representative Signature: ________________________

Date: _______ Year ___ Month ___ Day

Legal Representative's Contact Number: ________________________

Mobile Number: ________________________

Investigator Signature: ________________________

Date: _______ Year ___ Month ___ Day

Investigator's Work Telephone: ________________________

Mobile Number: ________________________

Contact Number of the Ethics Committee Office,

The First Affiliated Hospital of Henan University of Chinese Medicine:

0371-66285929
